# Investigating Sex-Based Neural Differences in Autism and Their Extended Reality Intervention Implications

**DOI:** 10.3390/brainsci13111571

**Published:** 2023-11-09

**Authors:** Rehma Razzak, Joy Li, Selena He, Estate Sokhadze

**Affiliations:** 1 Department of Computer Science, Kennesaw State University, Marietta, GA 30060, USA; rrazzak@students.kennesaw.edu (R.R.); she4@kennesaw.edu (S.H.); 2 Department of Software Engineering and Game Development, Kennesaw State University, Marietta, GA 30060, USA; joy.li@kennesaw.edu; 3 Department of Neurology, Duke University School of Medicine, Durham, NC 27710, USA

**Keywords:** autism, Autism Spectrum Disorder, brain activity, neurofeedback, extended reality

## Abstract

Autism Spectrum Disorder (ASD) affects millions of individuals worldwide, and there is growing interest in the use of extended reality (XR) technologies for intervention. Despite the promising potential of XR interventions, there remain gaps in our understanding of the neurobiological mechanisms underlying ASD, particularly in relation to sex-based differences. This scoping review synthesizes the current research on brain activity patterns in ASD, emphasizing the implications for XR interventions and neurofeedback therapy. We examine the brain regions commonly affected by ASD, the potential benefits and drawbacks of XR technologies, and the implications of sex-specific differences for designing effective interventions. Our findings underscore the need for ongoing research into the neurobiological underpinnings of ASD and sex-based differences, as well as the importance of developing tailored interventions that consider the unique needs and experiences of autistic individuals.

## 1. Introduction

ASD is a complex neurodevelopmental disorder, the symptoms of which include persistent deficits in social communication and interaction, as well as restricted and repetitive behaviors. These criteria are outlined in the Diagnostic and Statistical Manual of Mental Disorders, Fifth Edition (DSM-5) [1]. The range and severity of these symptoms can vary widely among individuals, as detailed in Table 1 [2,3]. The prevalence of ASD has increased significantly in recent years, with the Center for Disease Control (CDC) reporting that 1 in 36 children and approximately 2.21% of adults in the United States are affected by ASD [4]. Considering these statistics, the role of early diagnosis becomes increasingly critical. Early diagnosis not only paves the way for more effective, tailored treatments but also improves long-term outcomes for diagnosed individuals. Moreover, early diagnosis can have further-reaching implications, including the potential for broader societal benefits through more effective interventions [5]. In this context, psychometric tools specifically designed for early ASD diagnosis have already been developed, further enabling targeted and effective therapies [6].

Unfortunately, the challenge of diagnosing ASD becomes particularly evident when considering the varying prevalence rates of ASD between males and females. While ASD is more common in males than in females, with a male-to-female ratio ranging from 4:1 to 2:1 in most studies, women often face unique diagnostic hurdles. They frequently receive their ASD diagnosis later in life compared to men and are frequently misdiagnosed with other disorders [7]. This disparity can be attributed to a lack of understanding of ASD symptomatology in both sexes, inadequate diagnostic tools, and underlying genetic and cultural factors [8,9]. Additionally, ASD symptoms in women are often more complex, and some ASD symptoms may not show until later. In women with ASD, some symptoms that have been observed include sensory sensitivity, issues with executive function (though this can also be seen in autistic men), camouflaging, and emotion regulation [10,11]. Further evidence looked at how ASD and other mental health symptoms change in boys and women as they grow from pre-teens to young adults [12]. Researchers found that the greatest changes regarding the occurrence of ASD symptoms happened in adolescence; boys with ASD tend to have more repetitive behaviors and struggle more with change than women. However, as women with ASD grow older, they start to struggle with change as much as boys do.

**Figure 1:**
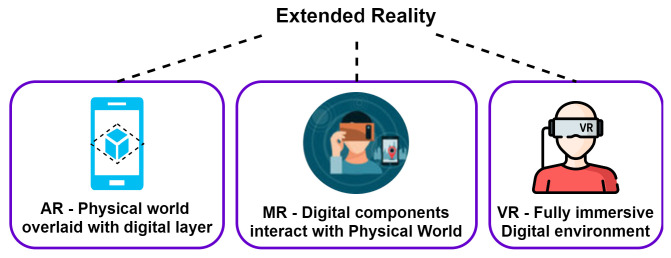
XR allows us to interact with the physical world via different mechanisms. XR technologies often rely on head-mounted displays, sensors, and other hardware to create a sense of presence and immersion in the virtual world.

In response to the diverse manifestations of ASD, a growing body of research has been focused on discerning the biological sex differences in both ASD presentation and brain activity, with the aim of devising tailored interventions utilizing cutting-edge extended reality (XR) technologies. XR is an umbrella term that encompasses all immersive technologies that augment or simulate the physical world, including virtual reality (VR), augmented reality (AR), and mixed reality (MR), as illustrated in Figure 1.

XR allows users to experience a computer-generated environment that is interactive and responsive to their movements, actions, and inputs. XR technologies have emerged as promising interventions for individuals with ASD [13,14]. They are shown to be highly effective in enhancing social communication skills, reducing anxiety in individuals with ASD, and helping parents and other staff (e.g., therapists, teachers) build intervention approaches tailored to the specific needs of the individual [15,16,17]. XR technologies have also been shown to target other areas that many with ASD can struggle with, such as enhancing/improvement of daily living skills and attention [18]. Such personalized approaches have the potential to enhance the efficacy of interventions and improve outcomes for individuals on the autism spectrum.

In the realm of identifying sex-specific distinctions in ASD brain activity, studies using functional magnetic resonance imaging (fMRI) have found that females with ASD exhibit greater activity in regions associated with social processing, such as the prefrontal cortex, compared to males with ASD [19,20]. In contrast, men with ASD show increased activity in regions involved in sensory processing, such as the visual and temporal cortices [21]. These findings suggest that the brain activity patterns associated with ASD may differ depending on an individual’s biological sex.

By integrating insights from these distinct neural patterns with individual needs, XR-based interventions can be customized to address the unique challenges faced by individuals diagnosed with ASD.

### 1.1. Methodology

To achieve a multidisciplinary understanding of ASD and XR’s applicability, we adopted a rigorous approach to our literature search. We sourced articles from eight databases, each selected for its unique focus and breadth, to ensure a well-rounded review:

- PubMed: PubMed: Specializing in medical sciences, this database was instrumental for acquiring the literature on the diagnostic and neurological aspects of ASD.
- IEEEXplore: Selected for its extensive collection of technology-related publications, particularly those focused on the application of VR, AR, and MR in ASD.
- Wiley Online: Contributed to a well-rounded review with its comprehensive range of subjects, including health, physical sciences, social sciences, and the humanities.
- MDPI: Particularly useful for its focus on extended reality (XR) technologies and as a supplementary source for articles not available in other databases.
- Frontiers: An open-access platform that publishes peer-reviewed articles across multiple disciplines, including science and technology, medicine, and the humanities.
- Elsevier, Springer, Semantic Scholar: These multi-disciplinary databases provided a broad scientific backdrop for our review.

To ensure the quality and relevance of the studies included in our review, we meticulously examined the following sections of each paper: Abstract, Methodology (including any experimental sub-sections), Results, and Discussions/Conclusions. These sections were chosen for their capacity to offer a concise yet comprehensive understanding of each study’s objectives, methods, outcomes, and relevance to our topic. We also employed Google Scholar to identify any articles that might have been missed in the initial database searches.

We extended our search to include studies that spanned the age spectrum, focusing on both children and adults with ASD, to offer a more comprehensive view of ASD throughout the lifespan. The inclusion criteria were as follows:

- The research must focus on at least one of the following key terms: ASD, brain structure, brain function, sex differences, or XR technology interventions.
- The studies must present empirical data relating to XR-based ASD interventions or brain activity. For the purpose of this review, “empirical data” refers to quantitative or qualitative information collected through observation or experimentation. This includes, but is not limited to, randomized controlled trials, observational studies, and validated surveys or questionnaires.
- The studies should provide some detailed participant characteristics, including, but not limited to, age, sex, and diagnosis criteria met. This also extends down to their methodology in terms of procedure, type of XR technology used, and equipment.
- The studies should offer significant findings—whether positive or negative—pertaining to the interventions or brain activities under investigation. The studies that had inconclusive findings were excluded.

Our search also focused on articles published between 2010 and 2023. The choice to focus on the literature published between 2010 and 2023 was deliberate, aiming to capture the most recent and relevant advancements in ASD research and XR technologies. While our search criteria initially spanned from 2000 to 2023, we found that the majority of the pertinent studies were published after 2010. This timeframe not only encompasses significant technological advancements in VR, AR, MR, and XR but also reflects shifts in the diagnostic criteria for ASD, such as the transition from DSM-IV to DSM-5 in 2013. Moreover, this period has seen a growing academic interest in exploring gender differences in ASD. It is worth noting that we did include a few seminal papers published prior to 2010 for their foundational insights. Our search also employed the following key terms:

- Autism Spectrum Disorder (ASD)
- ASD brain activity
- XR and ASD
- Virtual reality and autism
- Augmented reality and autism
- ASD in women

**Figure 2:**
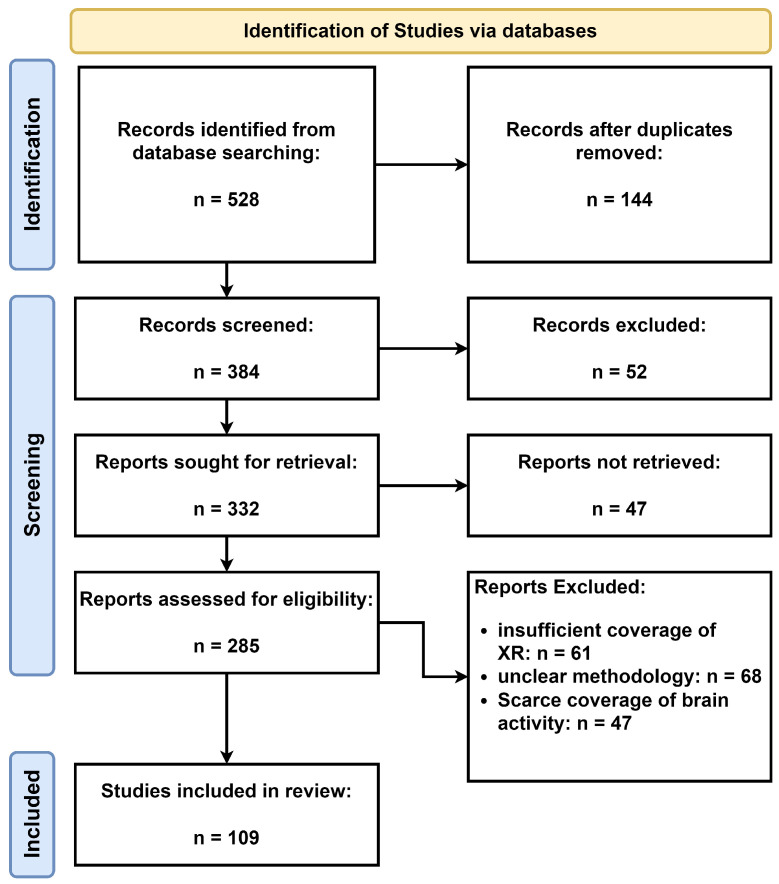
The PRISMA flow chart [22].

Figure 2 presents our process of using the Preferred Reporting Items for Systematic Reviews and Meta-Analyses (PRISMA) approach [22]. We identified a total of 528 records through database searching. The high number of duplicates removed (384) can be attributed to the multi-disciplinary nature of our search, leading to the same articles appearing in different databases. Additionally, some articles had multiple versions, such as conference papers that were later published in journals. After duplicates were removed, we were left with 144 records for screening. After these records were screened, resulting in the exclusion of 52 records that did not meet our inclusion criteria. We assessed 285 reports for eligibility and excluded 61 due to insufficient coverage of XR, 68 for having an unclear methodology pertaining the XR-based intervention for ASD, and 47 for scant coverage of brain activity across sexes.

This paper aims to enhance understanding of the neural mechanisms underlying ASD, with a specific focus on differences based on biological sex. We also delve into the potential of XR technologies for delivering personalized interventions tailored to these differences. Our contribution lies in shedding light on the neurological aspects of ASD in females—a demographic often underrepresented in research—and in evaluating the efficacy and impact of XR-based interventions.

By delving into these differences in brain activity patterns and the potential of XR interventions, our work contributes to the understanding of the complex neurological aspects of ASD. It also informs the development of more effective, tailored interventions. Our work offers valuable insights into the neurological aspects of ASD in women, a group often underrepresented in diagnoses and who present distinct traits compared to men. Additionally, we present detailed use cases of XR-based interventions for ASD, evaluating their applicability, usefulness, and potential impact, thereby contributing to the ongoing research in this field.

Throughout this paper, we use the terms ’male’ and ’female’ to refer to biological sex, focusing on the differences between individuals assigned as male and female at birth. We acknowledge that ’gender’ can encompass a broader spectrum of identities, but our study primarily examines sex-based differences in brain activity patterns among individuals with ASD. The reporting of this systematic review was guided by the Preferred Reporting Items for Systematic Review and Meta-Analysis (PRISMA) Statement [22].

### 1.2. Paper Organization

The remainder of this paper is organized into seven sections. Section 2 reviews the related works. Section 3 explores the distinct brain regions and organs associated with ASD in males and females. Section 4 examines various XR applications. Section 5 and Section 6 offer the discussions and limitations of the study, respectively. Section 7 concludes the paper.

## 2. The Related Literature

ASD presents a complex landscape of symptoms and deficits that vary across individuals. ASD is a wide spectrum disorder, so it is difficult to characterize individuals with a single stereotype. Each individual may have different deficits, or different combinations of deficits. XR technologies are increasingly recognized as a viable platform for such customization. This heterogeneity also underscores the need for research that differentiates between males and females, as emerging studies have revealed important insights into the variations in symptoms between the two. To comprehensively address these dual facets of research, this section is partitioned into two subsections: The first evaluates the practicality of employing XR-based interventions for ASD, while the second scrutinizes the variations in neural activity between males and females associated with ASD.

### 2.1. Feasibility of XR-Based Interventions for ASD

As summarized in Table 2, various reviews have explored the feasibility of XR-based interventions for ASD. One such study focused on how XR has improved Telehealth for those with ASD by reviewing publications from PubMed, Web of Science, and Cochrane Library between 2010 and 2022 [23]. After reviewing 112 articles, the authors found that VR and AR had the most qualitative and quantitative studies. Following XR-based interventions, there were notable improvements in the following areas:

- social interaction
- communication and speech
- emotion recognition and control
- daily living skills
- problem behavior reduction
- anxiety symptom reduction
- insomnia control

The above improvements were found from analyzing 14 randomized controlled trials, 26 non-randomized controlled trials, and 72 single group studies.

Similar findings were found by Mubin et al. [26], as several use-cases of XR that were discussed revealed an eagerness of children with ASD to learn through XR. Most of the reviewed studies examined by the research team demonstrated sufficient evidence for XR to assist autistic children. Although their coverage on the topic is relatively brief, the sentiment regarding XR’s high potential for ASD intervention remains consistent—that there is a positive outlook on its efficacy and applicability. However, the article does highlight the need to develop reliable validation in future studies to state that VR can effectively complement the traditional interventions.

With regards to VR specifically, several surveys have explored the feasibility of using it in ASD interventions. One systematic review, conducted by Mesa-Gresa et al., searched both clinical and technical databases. The findings suggest that VR-based treatments have led to improvements in at least one common ASD deficit in a majority of the studies. However, only 10 of these studies reported statistically significant improvements [39]. Regarding the technical aspects of VR, one area that is still an active research gap is guidelines—specifically the design guidelines for VR systems; These design guidelines are not always well-established, which can impact the overall design and reliability of the VR application. The existence of well-established guidelines for designing VR systems tailored for those with ASD was discussed [38]. The researchers found that most guidelines are derived from subjective observations and are highly task-dependent, raising questions about their generalizability, especially for adults with ASD. A different perspective summarizes the current evidence-based VR applications for children with ASD, particularly in the areas of social communication, emotion recognition, and speech [36]. While the authors acknowledge the capabilities of current VR technology for ASD interventions, they also point out caveats such as the importance of content and activity design, which are not often clearly defined or talked about in detail. Upon examining other studies, the authors found that the VR learning content used focused on situations that were too restrictive, and the skills that were taught were procedural and strongly rule-based with little emphasis on handling unpredictable social scenarios. Thus, it is also crucial to ensure that XR-based approaches undergo a strong validation process through well-designed evaluation processes [24]. Some of the studies lack this clarity, casting doubt on their overall validity due to inconsistencies in how these technologies are conceptualized [40].

In regards to AR-based interventions, similar issues are still apparent. A review of mobile AR noted that many of the existing mobile AR applications for those with ASD are not considered very practical due to inconsistent functionality [32]. There were also very small numbers of participants for the studies, and the reviewed studies were mainly conducted on children [31]. Other issues that were highlighted in the examined studies included: research design issues, no generalizability, no use of control groups, short intervention time, and participants not being differentiated by age. The main issue to be addressed, in this case, is the research design methodology and sample. This was also confirmed by Cavus et al. [33]. Further experimental studies are needed to establish the validity of AR interventions for individuals with ASD.

An article reviewed both AR and VR technologies for ASD in detail [34]; however, it focused more on utilizing them for teaching environments, which is out of scope for our purposes. The article provides a detailed review of AR/VR educational systems oriented to children with ASD. The authors obtained 24 research papers on the topic from 2017 to 2022, with AR being used in 14 of the studies and VR being used in 10 of them. However, half of the studies the authors examined did not mention what platform(s) were used. Interestingly, the Kinect platform was used in seven previous investigations, representing 29.17% of the total.

Turning to the broader scope of XR, the effectiveness of these interventions remains a subject of debate. One review included seventeen articles across five databases aimed to assess the effectiveness of XR interventions in enhancing social skills among autistic children [27]. Unfortunately, the study quality varied greatly, with a majority of studies being relatively low quality. Because of this, the study concluded that there is insufficient evidence to assert that the effects are generalizable, sustained, or even significant to the autistic population. These congruent findings are shared in other studies as well [28,35]. Specifically, the researchers found that the majority of the studies they reviewed had small sample sizes, no control groups, and no follow-up periods to measure the long-term effects of XR-based interventions. Most of the studies that were examined did not address XR-based interventions for higher-support need individuals, making it difficult to draw conclusions about XR’s effectiveness.

Building on the topic of XR, a comprehensive review analyzed 66 empirical studies focused on XR interventions for individuals with ASD [29]. The review scrutinized these studies through the lens of three key design aspects: autonomy, human–computer interaction, and the sense of presence. The majority of these studies (81.82%) employed VR as their primary tool, followed by AR in 16.67% of the cases and mixed reality in 1.52% of studies. The choice of XR platform was closely tied to the study’s technical features, affordances, and overall objectives. Interestingly, the review highlighted that the current XR interventions often lack adaptability; the scenarios presented do not adjust based on user performance. Additionally, the study pointed out that the existing XR systems fall short in facilitating natural, bidirectional conversations in lifelike situations—a gap that the future research could aim to fill. As for interaction methods, keyboards, mice, microphones, and touch screens were most commonly used. One significant gap identified was the limited research on the comfort levels of participants while using XR technology. Given that individuals with ASD may have heightened sensitivities to certain stimuli, understanding and measuring comfort could be crucial for the success of XR interventions, especially for those with special needs.

Overall, while XR applications show promising potential in many studies, the majority of them have low numbers of participants. In some cases, many studies did not include a control group at all, so there is no way to compare. Additionally, ASD exists on a spectrum, yet most studies seemed to include individuals who were considered high-functioning. As such, very few studies consider the severity of ASD symptoms and how it may affect XR-based ASD interventions. In fact, there have been some recent works that call for more inclusion of individuals on the spectrum who are diagnosed as level 2 or 3.

### 2.2. Differences in Brain Structures and Function Based on Sex for ASD

In addition to exploring the potential of XR for ASD, several studies have delved into the differences in brain structure and function between men and women with ASD. Table 3 provides a short overview of some of these findings. These studies primarily focused on resting-state functional connectivity in individuals with ASD, revealing distinct patterns in their brain connectivity [41,42,43,44,45]; they consistently report hyper-connectivity in specific brain regions, including the prefrontal cortex, paralimbic subsystem, and the striatum [41,42,43]. Conversely, hypo-connectivity is a common observation in the frontal and temporal lobes, amygdala, posterior cingulate cortex, postcentral gyrus, medial prefrontal cortex, inferior parietal lobule, and sensorimotor regions [44,45].

Biological sex was noted to also contribute to the etiology of ASD [19,46,53,54,55]. Some studies found similarities in cognitive functioning between men and women with ASD [49,52]; others have reported differences in brain activity patterns [19,46]. Overall, the findings suggest that biological sex affects the neurobiology of ASD, but more research is needed to fully understand these differences [53].

Studies have also used advanced computational algorithms to diagnose ASD using electroencephalogram (EEG) data [50]. While these studies show promise in their ability to accurately detect ASD, they are limited by their small sample sizes and the need for further validation.

Finally, longitudinal studies have examined the development of neural structure and function in children with ASD, revealing differences in cortical thickness and brain activity patterns compared to typically developing children [47,56]. These studies provide insights into the neurobiological mechanisms underlying ASD and have the potential to inform early interventions.

### 2.3. Remarks

Unlike some previous surveys, our review offers distinct contributions that set it apart:

- A critical evaluation of the existing research, complete with the identification of gaps and suggestions for future work.
- A comprehensive overview focusing on the specific brain regions most impacted by ASD.
- Novel insights into the sex-specific differences in the neurological manifestations of ASD.

## 3. Divergent Neurological Correlations in Males and Females with ASD

ASD manifests through a complex interplay of neurological factors, affecting various brain regions differently in individuals. Table 4 offers a concise overview of these affected brain regions.

Atypical brain development is a consistently reported finding in individuals with ASD, with the frontal and temporal lobes being the most commonly affected regions. The prefrontal cortex, which plays a vital role in social cognition and decision-making, is often reported to be impaired in individuals with ASD, affecting cognitive functions such as planning, problem-solving, and social behavior. The temporal lobe, responsible for language processing, memory, and social cognition, is reported to be affected in individuals with ASD. Other organs, such as the bilateral temporal lobe were noted to show significantly higher volumes in those with ASD compared with neurotypical individuals [57]. Other brain regions affected by ASD include the parietal lobe—involved in sensory processing and spatial awareness, and the cerebellum—involved in motor coordination and timing [7].

The earlier research, such as [45], has identified distinct brain activity patterns in ASD individuals across multiple regions. These include the right superior temporal sulcus—involved in processing social cues, and the right inferior and middle frontal gyri, which are essential for attention and social cognition. Variations in brain activity were observed in the bilateral cerebellar crus, right insula, and right postcentral gyrus, each responsible for motor control, emotional processing, and touch sensation, respectively. Earlier findings by [47] also found a significant enlargement of in both cerebral gray and white matter in toddlers with autistic disorder, with the most severe enlargement occurring in the frontal, temporal, and cingulate cortices. Women with ASD also showed a more pronounced abnormal growth profile in more brain regions than men with the disorder. The emerging research has begun to unravel more sex-specific neurological differences in ASD [19]. While findings are still inconclusive, there is growing evidence that brain regions associated with ASD differ between men and women, and these affected brain regions often have different nuances that set them apart from typically developing individuals [19,59].

The further research provides more evidence of there being notable observations of how ASD presents in women. Some studies suggest that women with ASD exhibit different patterns of brain involvement than men with ASD. Women with ASD may show more severe impairments in social communication and different patterns of brain connectivity. Both the frontal and temporal lobes may be particularly affected in women with ASD, similar to what is reported in men with ASD [58]. However, other research reported different findings, suggesting that women with ASD may have greater impairments in the parietal lobe, which is involved in spatial processing, attention, and sensory integration. One study found that women with ASD showed increased gray matter volume in the temporal lobe, while another study found that women with ASD showed increased activity in the frontal lobe during a social perception task, suggesting that women with ASD rely more on frontal lobe processes for social perception compared to men with ASD [59]. Women with ASD who carried a greater number of ASD-associated risk alleles also showed greater functional connectivity between the subcortical brain areas, which are important for motor learning [69].

It is important to note that research on the neurobiology of ASD in women is still relatively limited, and further studies are needed to clarify the specific patterns of brain involvement in this population [57]. Thankfully, new studies continue to find interesting insights into how brain anatomy differs by sex for those with ASD. Lai et al. [65] found that autistic women had heightened ventromedial prefrontal cortex self-representation, which was correlated with camouflaging. In contrast, intellectually able autistic women showed a lack of differences in neural responses [65]. This observation aligns with the findings of Mo et al., who reported no consistent neural activity variations between males and females, although the right inferior frontal-occipital fasciculus and the corpus callosum showed statistical significance [76].

Women with ASD and men with ASD have also exhibited notable differences in other brain organs. Supekar et al. identified distinctions between the two groups in the bilateral middle and superior temporal gyri and the dorsal parietal cortex [60]. These notable differences were mentioned by Walsh et al., who reported that the amygdala showed more atypical development in autistic women [61]. As such, the research regarding the brain structure changes in ASD suggests more atypical brain structure and function in women with ASD, as large regions showed abnormal growth, with the growth being more pronounced in women. Autistic women also had higher connections in the precuneus, cerebellum, and dorsal frontal cortex [7].

An interesting observation is the impact of XR interventions on the brain activity of autistic individuals. One study reported that VR social cognition training led to changes in brain activity in autistic individuals, specifically in the left inferior frontal gyrus and left superior parietal lobule [66]. Although the study’s sample size was small, their findings provide strong evidence of XR’s applicability to autistic individuals. It was found that women and males with ASD differ in the organization of cortical and subcortical motor systems. However, the severity of ASD symptoms was not related to several brain regions in either men or women, including the fusiform gyrus, amygdala, and insula regions [70]. In contrast, the studies indicate that regions such as the right fusiform gyrus, fronto-parietal, and right ventromedial prefrontal cortex play significant roles in differentiating neural activity patterns between males and females in ASD [62].

Meanwhile, Cauvet et al. investigated the relationship between structural social brain networks and social cognition in women and men in relation to ASD and autistic traits in twins [67]. The study found increased thickness of the inferior frontal gyrus in participants with ASD, particularly in men. However, the association between social cognition and autistic traits did not differ significantly between the sexes.

A more conclusive finding indicated that autistic women displayed greater activity in the nucleus accumbens relative to autistic boys, as well as greater activity in the lateral frontal cortices and the anterior insula compared with typically developing women [63]. It was also reported that ASD women showed markedly reduced response versus typically developing women, particularly in sensorimotor and frontal regions [68].

While the aforementioned studies offer insights into specific brain regions associated with ASD, it is crucial to consider broader techniques that provide a comprehensive view of the brain’s activity. One such technique that has gained prominence in the study of ASD is quantitative EEG (QEEG). QEEG is a non-invasive tool that generates color-coded brain maps, offering a visual representation of the brain’s activity [81,82,83].

Numerous studies have underscored the significance of QEEG in pinpointing specific brain regions associated with ASD. For instance, an absolute delta deficit was highlighted over the frontal and central regions and theta excesses over the frontal, temporal, and posterior regions in an ASD sample [84]. Another study further elucidated the functional abnormalities within the thalamus, hippocampus, caudate nucleus, posterior cingulate, supramarginal gyrus, and various occipital/temporal and parietal cortical regions in individuals with ASD [85]. Contrarily, differences were not observed in the absolute power of delta frequency bands across the frontal, temporal, and occipital regions [86]. However, Dimitrov et al. reported elevated delta, theta, and beta levels, coupled with reduced alpha levels in children with ASD [87]. This was also supported by QEEG results showing increased delta–theta activity in the frontal region of the brain [88]. Nevertheless, QEEG remains a method with significant promise not only for deepening our understanding of ASD but also for its potential in broader applications and areas. This multi-faceted utility sets the stage for the technique’s evolving role in the field.

Beyond mere identification, QEEG’s potential as a biomarker for ASD was proposed, emphasizing its capability to discern clinical groups from typical individuals and those with ASD with high precision [89,90]. Such findings underscore the technique’s promise in elucidating the specific brain regions implicated in ASD, paving the way for targeted interventions and therapies.

In summary, the breadth of the research underscores the intricate neurological landscape of ASD, emphasizing the nuanced differences across men and women. Recognizing these disparities is paramount, as it paves the way for tailored therapeutic approaches that resonate with the distinct neurological signatures of each individual with ASD. As we continue to deepen our understanding, it becomes increasingly evident that a one-size-fits-all approach is insufficient; instead, a personalized strategy, informed by these neurological insights, is the key to effective interventions.

## 4. XR Use Cases for ASD

In this section, we explore various applications of XR for ASD and their outcomes. The purpose of highlighting these use cases is to demonstrate the wide applicability of XR in helping with various areas of ASD and to provide evidence for the effectiveness of XR-based interventions. Table 5 provides a general summary of these use cases in terms of areas of improvement, the method used, and the specific use case of XR.

### 4.1. Social Skills

XR technologies have shown significant promise in enhancing the social capabilities of individuals with ASD. A common thread across various studies is the use of XR to improve the understanding and recognition of facial expressions, body language, and other non-verbal cues, which are often challenging for those with ASD.

A popular use case for XR includes enhancing the social capabilities of those with ASD. One of the earliest examples implemented a real-time facial analysis system [99]. Although not explicitly an XR technology, its core principles resonate with XR’s objectives. The system, equipped with a Samsung ultramobile computer and a Logitech camera, was adept at tracking 24 facial feature points. It could then predict a person’s cognitive state based on these facial and head movements. A standout feature was the “Emotion Bubbles” visualization, which intuitively represented emotions as bubbles, their size indicating the emotion’s intensity. This immediate feedback mechanism aimed to enhance the user’s understanding of real-time emotions. Tested on adolescent boys diagnosed with ASD, the results were encouraging, underscoring the potential of such real-time feedback systems in bridging the gap between early technological interventions and modern XR applications for ASD.

Building upon these foundational technologies, more immersive environments were developed to foster social interaction. For example, an interactive environment named “Lands of Fog” was co-created with children with ASD. Projected on a 6-meter diameter circle, the environment emphasized exploration and teamwork, encouraging interaction with diverse terrains [96]. Along similar lines, Kinect was employed for role-play scenarios, enabling trainers to manipulate 3D virtual characters in real-time. This innovative method provided children with ASD the opportunity to learn the nuances of body language through portrayed social interactions [93]. These advancements signify the growing potential of XR technologies in enhancing social interactions for individuals with ASD.

Continuing the focus on social capabilities, the recognition of nonverbal social cues has also gained prominence as a pivotal research area. Various applications are now at the forefront of aiding those with ASD in this aspect. For instance, an AR-based Video Model Storybook (ARVMS) was designed to blend traditional storybook elements with AR on the Vuforia mobile vision platform [92]. Further extending the scope, an AR application using Unity and Kinect was developed to specifically enhance facial expression comprehension [94]. Taking a different approach, Li et al. introduced “FaceMe,” an AR-based game accompanied by tangible toolkits. This game targets emotional development in children and was positively received in the initial studies [95]. In the realm of VR, Rosenfield et al. developed “Bob’s Fish Shop”—a game that allow users with ASD to engage in social interactions within a pet-themed Unity 3D environment for the Oculus Rift [97].

Expanding the applications of XR technologies beyond social cues and interactions, there has been a marked advancement in tools aimed at enhancing communication for non-verbal individuals with ASD. A noteworthy example is HoloType-CR, an MR application designed for the HoloLens 2 by Alabood et al. [91]. Building upon their previous work with HoloType, a typing training application for the same platform, the researchers have now evolved it into a cross-reality tool. This advanced version facilitates communication between minimally verbal persons (MVPs) and their (communication and regulation partners (CRPs) by allowing them to interact in a shared virtual environment.

### 4.2. Life Skills and Daily Activities

While much of the research has focused on the social and communication aspects of ASD, XR technologies have also been applied to another critical domain: daily living tasks. Given that individuals with ASD often face challenges in various aspects of day-to-day life, this area remains an active field of study. Early endeavors took simple yet impactful steps. For instance, VR was utilized to teach the intricacies of street-crossing to children with ASD, as seen in a study that employed the ProVision 100 system [102]. Interestingly, the study revealed varied perceptions of the virtual environment among participants. While one child navigated the 3D space effectively, the other perceived it as a two-dimensional flat screen. Building on such foundational work, more advanced tools such as the Oculus Rift headset have also been employed to teach street-crossing safety skills [103]. Utilizing a series of video clips that depict both safe and unsafe pedestrian behaviors, this approach trained children to discern appropriate actions for crossing streets. The immersive VR environment offered a realistic yet risk-free setting for this crucial life skill.

Beyond the prominent focus on social interactions, there are unique ventures in applying XR technologies for different outcomes. One such early venture is Astrojumper, a VR game developed in 2010. Set in a space-themed environment, the game challenges children to dodge virtual objects through physical movements. Created using OpenSceneGraph and OpenAL and projected on a three-sided CAVE, Astrojumper demonstrated the potential for XR to improve body coordination and activity levels [104].

Expanding the scope of XR applications, fine motor control, another vital daily living skill, can be difficult for individuals with ASD. These challenges can manifest in varying degrees and across different motor functions. One innovative solution is CheerBrush, an AR coaching system designed by Zheng et al. to improve toothbrushing skills [105]. The system uses a virtual avatar to provide a guided, step-by-step toothbrushing experience, adapting the difficulty level as needed. It also features a wearable E4 sensor to monitor stress levels during the activity, providing valuable insights into the coaching’s effectiveness. The initial studies involving both children with ASD and typically developing children were encouraging, showing not only improved brushing techniques but also reduced stress levels. Parents also reported finding the system beneficial.

While a plethora of XR interventions focus on enhancing daily living skills in children with ASD, resources for adults in this domain are comparatively less common. Nevertheless, this is an active area of research, catering to unique challenges adults with ASD face. One such innovation is InterViewR, a mixed-reality platform that was tailored to prepare these individuals for job interviews. By fusing VR with wearable smart technology, InterViewR provides biofeedback based on both behavioral and physiological metrics. This biofeedback mechanism aids in honing interview skills, demonstrating the adaptability of XR technologies in addressing life-stage-specific challenges [106].

Continuing on the theme of adult-specific challenges, driving emerges as an essential skill warranting specialized attention. A significant contribution in this domain comes from Wade et al., who developed the VR adaptive driving intervention architecture (VADIA). The system utilizes real-time gaze data to adapt a meticulously crafted 3D virtual city, offering a range of driving scenarios. These scenarios are categorized into trials such as turning, merging, and speed maintenance and are further organized into graded assignments with immediate performance feedback. A pilot study involving 20 adolescents with ASD demonstrated the system’s potential, underscoring its effectiveness in enhancing adult-specific daily living skills [107]. The subsequent research continues to unravel the complexities of driving performance for individuals with ASD, pinpointing challenges in areas such as lane maintenance and speed management. Despite these challenges, the advent of XR technologies has offered tailored solutions, helping people with ASD build confidence in their driving abilities.

Another aspect of daily living is mental well-being. Anxiety, in particular, is a significant concern among individuals with ASD. While not a diagnostic criterion for ASD, high levels of anxiety are commonly reported among this population. The triggers for such anxiety can vary, from sensory sensitivities to uncertainty in social situations. Aiming to address this, there are emerging interventions focused on anxiety management and relaxation. One such initiative is the development of “Magic Bubbles”, an MR application created for the MiRERA platform. Designed to aid relaxation, the application employs the HTC Vive Pro headset and Zed Mini camera. Using Unity3D and Google Resonance Audio among other tools, it strikes a balance between technical sophistication and a user-centric design. An initial pilot study has shown promise in helping children with ASD manage their anxiety levels [101].

### 4.3. Concentration

Another area of interest that is an active area of research is concentration. This focus aligns well with the common observation that many individuals with ASD are visual learners, which offers fertile ground for XR interventions.

One notable XR application that aids attention management is Mobis, as introduced in a study by Escobedo et al. [108]. Mobis seamlessly blends the digital and physical worlds by overlaying digital content on physical objects. Its main goal is to enhance engagement during therapy sessions for children with autism. Mobis features a multifaceted architecture, with a core therapy manager that customizes the therapy experiences. Teachers can manually adjust it while it also adapts autonomously based on real-time progress assessment. The system employs the ambient notification system (ANS), which runs on Android smartphones and captures object images triggered by accelerometer events. These images are processed by the ANS server for object identification. Mobis also includes a tag manager that allows teachers to create a database of object images associated with specific tags and therapies. This system enhances attention and engagement among children with ASD, as evidenced by its positive impact, increasing on-task time by 20% and improving selective attention by 62%.

Another XR application addressing attention management is MARA, proposed by Wang et al. [109]. Focusing on autistic adults, the authors categorize attention into three types: selective (focusing on a single task in the presence of distractions), sustained (maintaining focus for an extended period), and spatial (distributing and reallocating focus across spaces). Developed using Unity3D and the Vuforia AR kit, MARA provides an interactive experience that includes real-world and augmented perspectives filled with virtual stimuli. The users select a physical object as a focal point, with the real-world view intermittently replaced by an augmented perspective. As users progress, they are provided feedback on their anxiety, motivation, and distraction levels. The user-friendly interface of MARA also ensures seamless object tracking and user interaction.

In essence, XR applications such as Mobis and MARA exemplify the potential of immersive technology in addressing concentration challenges among individuals with ASD. By bridging the digital and physical realms, these applications offer tailored experiences that cater to the unique learning styles commonly found in individuals on the autism spectrum.

### 4.4. Neurofeedback

The emerging research suggests promising avenues for integrating XR technologies into neurofeedback interventions for ASD. This is a burgeoning field, with initial findings pointing to XR-based neurofeedback as a novel approach for ASD intervention. A few recent notable examples of such XR-based neurofeedback interventions are discussed below.

Lu et al. developed a VR-based approach to create interactive and immersive environments aimed at enhancing users’ attention. This approach utilized the α (alpha) band power in the parieto-occipital regions of the brain as a neurofeedback index for attention. The study observed a significant reduction in α band power among individuals with ASD, indicating improved attention. The task performances also demonstrated a 6.44% enhancement [110].

Similarly, Amaral et al. also employed VR. More specifically, they employed a brain–computer interface (BCI) paradigm within a VR context to train social cognition skills in individuals with ASD. This BCI training spanned seven sessions over four months and involved participants identifying objects of interest based on the gaze direction of an avatar in a virtual environment. EEG data, specifically the P300 component, were used to detect neural signatures of attention processes [98]. Although the primary outcome measure (rate of automatic responses to joint attention cues) did not exhibit significant changes, secondary neuropsychological measures showed improvement.

In contrast, Lyu et al. introduced an innovative AR-based approach to enhance social and attentional capabilities in children with ASD. Their mobile neurofeedback training (NFT) game, “Eggly”, targeted preschool children aged 30 months to 6 years. Eggly incorporated AR technology and a consumer-grade EEG headband to deliver a unique gaming experience centered around helping characters collect eggs, requiring players to exercise empathy and social collaboration skills. The brain activity, specifically the mu rhythms linked to social cognition, was monitored. Mu suppression levels were used as a measure of social skill function and integrated into the game as gamified feedback. Five children with ASD participated in the study, reporting positive feedback on the game’s setup and usability [100].

While these studies represent a limited sample, they provide compelling evidence of the potential for integrating neurofeedback with XR technologies. Given the ongoing research in neurofeedback and XR, combining these approaches could yield even more targeted and effective ASD intervention treatments.

## 5. Discussion

In this review, we aimed to provide a holistic view of the impact of ASD on the brain, with a particular focus on sex-specific neurological differences and the potential of XR for personalized interventions. Through this multi-faceted lens, our work offers what we consider to be a more nuanced understanding of the relationship between ASD and the brain. By “nuanced”, we mean that our survey delves into some of the more intricate aspects of ASD that are not always well-emphasized or thoroughly understood in the broader literature. Moreover, our focus on sex-specific differences in neurological manifestations of ASD provides a more individualized understanding of the disorder, which is crucial for developing targeted interventions.

Our findings support and extend the existing research by highlighting distinct neural activity patterns in ASD that vary between males and females. In women with ASD, we observed heightened activity in regions crucial for social processing, such as the prefrontal cortex, when compared to men with ASD. This finding suggests that women may utilize different neural pathways for social navigation, potentially contributing to their higher levels of empathy and improved social communication skills. Conversely, men with ASD exhibited increased activity in regions associated with sensory processing, reinforcing the importance of interventions tailored to the unique needs of men and women with ASD.

Building off this, our review also shed light on the promising potential of XR in ASD interventions. These technologies offer a controlled yet realistic environment for practicing social interactions and skill-building, and their immersive nature enhances user engagement, thus, increasing the efficacy of interventions. XR’s versatility means that they can target a wide range of ASD symptoms. Additionally, with the real time feedback provided by XR, XR can help individuals with ASD understand and correct their behaviors immediately. This enables them to transfer skills learned from XR into real world settings.

However, the field still has many avenues for further exploration. One crucial area that warrants additional investigation is the long-term effectiveness and generalizability of XR-based interventions. The future research should aim to examine the sustainability of acquired skills and their transferability to everyday settings. Another significant gap in the current literature is the under-representation of individuals with higher support needs in ASD studies, a demographic that the future research should aim to include more comprehensively.

## 6. Limitations

While our study offers valuable insights into the gender-specific neurological differences in ASD and the potential of XR technologies for interventions, it is essential to acknowledge several limitations that may impact the generalizability and comprehensiveness of our findings.

One limitation of our study is that it primarily focused on the biological neurological differences in ASD. The study did not explicitly consider individuals who identify outside the binary genders or those who are on the LGBTQ+ spectrum. This limitation may restrict the generalizability of our findings, as it does not fully encompass the diverse gender identities within the ASD population. The future research should prioritize inclusivity by exploring the experiences and neurological manifestations of ASD in individuals with diverse gender identities.

Our study also revealed a scarcity of studies that specifically addressed the inclusion of individuals with ASD that have high support needs. These individuals often face more profound challenges in daily living and may require more intensive interventions. As a result, our findings may not sufficiently inform the suitability and effectiveness of XR interventions for this particular subgroup within the ASD population. The future research should prioritize investigating XR interventions tailored to individuals with high support needs, recognizing the unique requirements and challenges they present. This would contribute to a more comprehensive understanding of XR’s applicability across the entire spectrum of ASD.

## 7. Conclusions

As we look ahead to future studies, gaining a more detailed understanding of the neurobiological aspects of ASD is crucial. This deeper comprehension not only enhances our grasp of the disorder but also serves as the cornerstone for the development of precise and effective treatments.

By acknowledging the impact of ASD on specific brain regions, the unique neurobiological differences, and exploring the potential of XR technologies, we are positioned to craft more effective and highly personalized interventions. This comprehensive approach not only holds the promise of improved outcomes for individuals with ASD but also represents a significant advancement in our ongoing quest to enhance the quality of life for those affected by ASD.

## Figures and Tables

**Table 1:** Three Levels of ASD. Adapted from [2].

Levels of ASD	Characteristics
Level 1: Requiring Support	Difficult social initiation Limited/unusual responses Difficulty switching activities Difficulty with organization
Level 2: Requiring Substantial Support	Marked deficits in social communication Social impairments with support Limited social initiation Difficulty maintaining conversation Inflexibility, difficulty with change Stereotyped/repetitive behaviors No interest in peers
Level 3: Requiring Very Substantial Support	Severe social communication deficits Abnormal nonverbal communication Little or no functional language Inability to initiate social interactions Extreme inflexibility, difficulty with change Stereotyped/repetitive behaviors Absence of interest in peers

**Table 2:** Related works regarding the feasibility of XR.

Author(s)	Topic	General Findings	Pros	Cons
Bertram et al. [24]	XR	CAT shows promise, lacks rigorous assessment	Covers multiple areas, provides VR use cases	No consideration for alternatives
Shoaib et al. [25]	XR	Benefits those with ASD, improves skills	Provides VR use cases	Limited tech coverage, insufficient research
Mubin et al. [26]	XR	XR improves behavior, attention, reduces stress in ASD	Covers XR use cases and weaknesses	No participant info, limited analysis
Chen et al. [23]	XR	XR improves ASD outcomes across numerous areas	Categorizes literature by XR tech type	Lack of info on hybrid interventions
Roberts et al. [27]	XR	Limited evidence on XR’s effectiveness for ASD children	Quality of XR-based studies, mentions of brain anatomy	Unclear effectiveness on older teens
Maran et al. [28]	XR	XR improves navigation and daily living skills for ID individuals	Wide age range of participants, varying degrees of ID	Small sample sizes, no control group
Gu et al. [29]	XR	XR platform usage tied to technical features and study traits	Review of XR technologies, high number of studies	No consistent quality check
Karagiannidis et al. [30]	AR	AR can help people with ASD learn new skills	Coverage of use cases	Very low number of studies
Wedyan et al. [31]	AR	Limited long-term research on AR’s effectiveness with autistic children	Proposes new AR system, strong evidence for AR usage	Small sample sizes, short study durations
Lian et al. [32]	AR	Mobile AR aids autism intervention to some degree	Discusses mobile applications of AR	Studies involved mostly children, small sample size
Cavus et al. [33]	AR	AR applicable for multiple fields and disabilities	Calls for more AR-based research	Limited search criteria
Huamanchahua et al. [34]	AR	AR/VR can enhance education, but usage is limited	Discusses AR and VR platforms used	Participants’ characteristics not discussed
Shahmoradi et al. [35]	VR	VR technologies had beneficial effects on reducing cognitive problems	Strong assessment of study quality	Very low number of studies, low numbers of women participants
Zhang et al. [36]	VR	VR improves social functioning, emotion recognition, speech, and language	Overview of popular VR products	Does not discuss applicability for adults with ASD
Glaser et al. [37]	VR	Inconsistencies in how VR is conceptualized	Need for clear VR definition	Does not address other types of XR technologies
Bozgeyikli et al. [38]	VR	VR design guidelines for ASD individuals rely on observation and lack generalizability	Covers VR system types, design considerations	Low number of participants
Mesa-Gresa et al. [39]	VR	VR-based treatment improves at least one common ASD deficit	Reviews clinical and technical databases	Limited insight into variations between males and females. Focus on ‘high performance autism’

**Table 3:** A short summary of studies relating to brain activity for individuals with ASD.

Author	Technique(s) Used	Pros	Cons
Supekar et al. [41]	fMRI (functional magnetic resonance imaging)	• Categorizes brain systems by function • Very large sample size of data	• No discussion on sample size characteristics
Alaerts et al. [46]		• Large sample size of women participants • Strong evidence of sex-based brain activity differences • Consideration of ASD symptom severity	• Discusses some brain organs without defining their functions
Schumann et al. [47]	MRI	• Strong potential for diagnosing ASD much earlier	• Outdated information regarding sub-categories of ASD
Ecker et al. [21,42,48]		Large sample size of adults with and without ASD Significant differences found in brain regions of both groups	• Exclusively used men • Very little variety in adult age range
Lai et al. [49]		• Large varied sample population • Wide age range	• Not all aspects of ASD in adults were examined in detail
Hull et al. [43]	rs-FMRI	• Comprehensive coverage of rs-FMRI, its applications and weakness	• No studies were found that equally represented men and women
Subbaraju et al. [44]		• New approach for extracting features to identify differences in women with ASD • Discusses several brain organs and their correlation with ASD	• Limited sample size for women
Grossi et al. [50]	EEG	• The proposed method achieved 92% accuracy • Comparison against multiple machine learning algorithms	• Limited sample size for the study
Buch et al. [51]	Machine Learning	• Unique contribution on how ASD is diagnosed • Large sample sizes used	• Emphasizes genes, but explanation regarding role is unclear.
Frazier et al. [52]	Data analysis from the Simons Simplex Collection. *t*-tests were also used for determining clinical characteristics among women.	• Large sample size of women participants	• Brain anatomy not discussed • Data analysis methodology unclear
Paaki et al. [45]	Regional Homogeneity (ReHo) Analysis	• Strong application in using ReHo for ASD analysis • Can support fMRI studies for analyzing ASD	• No distinction in regards to sex

**Table 4:** A summary of various brain regions and the related literature addressing them, along with the associated organs in that brain region. Some organs are associated with multiple regions and are indicated by an asterisk (*) symbol.

Brain Region(s)	Related References	Associated Organs
Temporal Lobe	[45,47,57,58,59,60,61,62,63,64]	Middle Temporal Gyrus (MTG)
		Amygdala
		Fusiform gyrus
		Hippocampus *
		Insula *
Frontal Lobe	[7,47,58,62,64,65,66,67,68]	Anterior cingulate cortex (ACC)
		Cingulate gyrus
		Dorsal anterior cingulate cortex (dACC) *
		Dorsolateral prefrontal cortex (DLPFC)
		Inferior frontal gyrus (IFG)
		Medial prefrontal cortex (MPFC)
		Prefrontal cortex
		Superior frontal gyrus (SFG)
Subcortical Structures	[69,70]	Basal ganglia
Cerebellum	[71,72,73,74,75]	Cerebellar vermis
Occipital Lobe	[64,76]	Visual cortex
		Lateral occipital cortex
Parietal Lobe	[7,62,64,66]	Superior parietal lobule
		Posterior cingulate cortex (PCC) *
Corpus Callosum	[76,77,78,79,80]	N/A

**Table 5:** Extended Summary of Studies on Areas of Improvement with Sub-Categories.

Areas of Improvement	Sub-Category	Citation	Method	Specific Use Case(s)
Social Skills	Nonverbal Communication	[91]	MR	Nonverbal communication
	Body Language and Facial Expressions	[92,93,94,95]	AR	Body language and facial expressions
	Social Interaction	[96]	MR	Collaboration
		[97,98]	VR	Social interaction and social cognition
	Emotions	[99,100,101]	- AR MR	Understanding Emotions Empathy Relaxation
Life Skills and Daily Activities	Visual Perception ^1^	[102,103]	VR	Street crossing
	Physical Well-being ^2^	[104,105]	VR AR	Exercise Improving fine motor skills
	Daily Living ^3^	[106,107]	MR VR	Preparing for job interviews Driving
Concentration	Task Engagement	[108]	AR	Therapy sessions
	Attention Management ^4^	[109]	AR	Attention management in terms of distraction
	Attention Enhancement ^5^	[110]	VR	Attention enhancement
Education	Special Education ^6^	[111,112]	MR AR	Helping special educators teach children with ASD Improving English Vocabulary

^1^ Individuals with ASD may have visual perception challenges affecting safe environment navigation, such as street-crossing. ^2^ Physical activities can help in enhancing both fine and gross motor skills, which are often a challenge for individuals with ASD. ^3^ With XR aiding ASD individuals in social skills, emotional regulation, and executive functioning, real-life tasks such as job interviews and driving become easier. ^4^ Although not considered in the core diagnostic criteria, attention problems are also common in ASD. ^5^ Attention enhancement can be crucial for those with ASD, as they often struggle with maintaining focus, especially in environments with numerous distractions. ^6^ The ’AReal-Vocab’ app demonstrates enhanced language acquisition in ASD students, crucial for their communication and social interaction. This also caters to the visual learning preferences of these students, further enhancing engagement.

## Data Availability

No new data were created or analyzed in this study.
